# Identifying early screening factors for depression in middle-aged and older adults: A cohort study

**DOI:** 10.1515/med-2025-1160

**Published:** 2025-03-19

**Authors:** Guanqun Chao, Lan Zhang, Zheli Zhan, Yang Bao

**Affiliations:** Department of General Practice, Zhejiang University School of Medicine Sir Run Run Shaw Hospital, Hangzhou, China

**Keywords:** depression, CHARLS, risk factor, middle-aged and elderly people

## Abstract

**Objective:**

This study aims to explore the current status of depression and related factors in middle-aged and elderly people in China using a cohort database with multi-year follow-up.

**Methods:**

The study population for this project was derived from the China Health and Retirement Longitudinal Study. Participants were divided into control and depression groups based on scores from the Center for Epidemiological Research Depression Scale. Continuous variables were compared using *t*-tests or Mann–Whitney U tests, while categorical variables were compared using chi-square tests. A multivariate logistic regression model was employed to evaluate factors associated with depression.

**Results:**

A total of 9,749 participants were included in the study. Correlation analyses revealed that age, body mass index, diastolic blood pressure, waist circumference, total cholesterol, uric acid, and length of sleep were significantly associated with depression (*p* < 0.05). Women exhibited a higher risk of depression in middle and old age compared to men (*p* < 0.05). An increase in waist circumference was associated with a decreased risk of depression (*p* < 0.05). Longer sleep duration and higher educational levels were also associated with a reduced risk of depression (*p* < 0.05). Unfavorable marital status and decreased frequency of alcohol consumption were found to increase the risk of depression (*p* < 0.05).

**Conclusion:**

In middle-aged and elderly individuals, women, low education level, and experiencing dissatisfaction in marriage are connected with a high risk of depression. On the other hand, increased waist circumference, moderate alcohol consumption, and longer sleep duration are connected with a low risk of depression.

## 1 Introduction

Depression is a common mental disorder caused by the interaction of biological, psychological, and social factors [1]. Depression is considered to be one of the main causes of disability due to the large number of lives lost, but there are still problems, such as non-response and partial response to antidepressant treatment [2]. As a mental illness, depression affects approximately 5% of the global population, and researchers believe that its underlying mechanisms involve cytokines, hormones, oxidative stress, and neuropeptides [3]. Studies have found that depressed patients are prone to elevated blood lipids, which is why depression is at increased risk of cardiovascular events [4]. Additionally, researchers have discovered that major depressive disorder is linked to age-related negative outcomes and overlaps with biological changes associated with aging [5]. This suggests that depression may be connected to the aging process, highlighting the urgent need for prevention and intervention strategies targeting middle-aged and elderly individuals.

There have been numerous studies conducted on risk factors for depression, but the results remain somewhat controversial. Specifically, when it comes to depression in older adults, there is a large body of research; however, the conclusions drawn from these studies are still unclear. In studies of veterans, age was found to be a negative predictor of depression, whereas secondary analysis found that age was a predictor of remission, so age is not just a predictor of depression outcome [6]. The depressive state is associated with physiological changes such as hypercortisolemia, activation of inflammation, and reduction of nutritional factors [7]. The study found that more than half of the elderly in Egypt have depression, and its risk factors mainly include female gender, unemployment, chronic disease, and low income [8]. In addition to this, living environment, social support, and autonomy can also influence depressive status, so studies have found that titles in nursing homes are more likely to be depressed, especially in low- and middle-income countries [9]. Depression is also affected by genetics, and studies have found that the heritability of depression is at 37%, and genetic imaging can explore brain abnormalities caused by genetic variations, which is used to test for depression [10]. It is evident that depression, particularly in relation to aging, has garnered significant attention worldwide. Consequently, research on depression in the elderly has become a prominent area of focus. At present, most of the research on depression focuses on adolescents and the elderly, and there are relatively few studies on middle-aged people. However, due to the current social state, life pressure, and work pressure, middle-aged people are the group of people who are in an abnormal mental and psychological state. With the process of an aging society, the elderly are a group of people who need care. For this purpose, we selected middle-aged and older people to carry out the study. This study aims to utilize a cohort database with multi-year follow-up in China to explore the current status of depression and its related factors in middle-aged and elderly individuals.

## 2 Method

### 2.1 Study population

The study population for this project was derived from the China Health and Retirement Longitudinal Study (CHARLS) [11]. The study comprised a representative sample of Chinese individuals aged 45 years and older, with baseline surveys conducted in 2011 and subsequent follow-ups conducted every 2–3 years. CHARLS received approval from the Biomedical Ethics Review Committee of Peking University and obtained informed consent from all participants.

### 2.2 Grouping

This study utilized the Center for Epidemiological Research Depression Scale (CES-D-10) to evaluate depression. The CES-D-10 has been widely employed to assess depressive symptoms in older adults and has demonstrated effectiveness [12,13]. Each item on this scale is rated on a scale of 0–3, with higher cumulative scores indicating greater levels of depression. Scores equal to or exceeding 10 are indicative of depressive symptoms [14]. Participants were categorized into control and depression groups based on their scores.

### 2.3 Questionnaire content

The study included participants’ education level (no formal education; did not finish primary school; Sishu/home school; elementary school; middle school; high school; vocational school; two-/three-year college/associate degree; four-year college/bachelor’s degree; master’s degree; doctoral degree/Ph.D.), marital status (married and living with spouse; married but not living with spouse temporarily for reasons such as work; separated, not living together as a couple anymore; divorced; widowed; never married), and alcohol consumption (more than once a month; less than once a month; none).

### 2.4 Relevant inspection indicators and inspection indicators

The study included several indicators such as age, gender, height, weight, waist circumference, blood pressure, blood lipids, uric acid, blood sugar, sleep duration, and more. Body mass index (BMI) is calculated as weight (kg) divided by height (m) squared.

### 2.5 Statistical analysis

All data were analyzed using SPSS 25.0. A *t*-test or Mann–Whitney U test was used for comparing continuous variables, while a chi-square test was used for comparing categorical variables. The *t*/χ^2^ tests were employed to assess the differences in demographics and characteristics between the two groups. A multivariate logistic regression model was utilized to evaluate the factors associated with depression. The mean of continuous data is presented as mean ± SD. Statistical significance was set at *p* < 0.05.


**Informed consent/Patient consent:** All participants signed informed consent at the time of participation.
**Ethical statement:** Approval for the original CHARLS was obtained from the Biomedical Ethics Review Committee of Peking University (IRB00001052-11015).

## 3 Results

### 3.1 Sample inclusion

**Figure 1:**
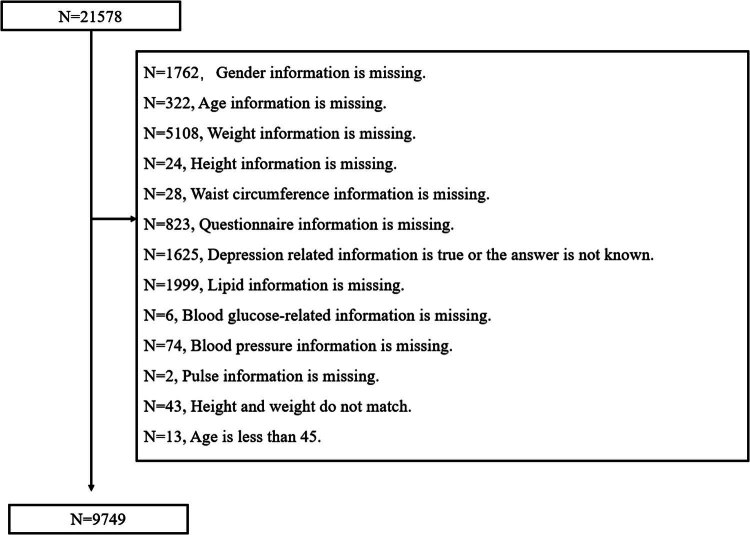
Flow diagram.

The databases from 2015 and 2018 were eventually combined based on the ID of the subjects, resulting in a total of 21,678 subjects. Invalid data, such as missing information on sex, age, questionnaire responses, biochemical indicators, blood pressure, pulse, etc., were subsequently removed. As a result, 9,749 participants were included in the final analysis (Figure 1).

### 3.2 Comparison of baseline data between the depression group and the control group

Participants aged 45 years or older were included, resulting in an average age of 66.57 ± 9.28 (Table 1). Notably, there was a significant difference in age between the depression group (66.89 ± 9.13) and the control group (66.38 ± 9.36) (*p* < 0.05). Additionally, the proportion of women in the depressed group was higher (62.7%), while the control group had a higher proportion of males (52.5%). This gender difference between the two groups was statistically significant (*p* < 0.05). Furthermore, questionnaire surveys revealed significant differences in education, marital status, and alcohol consumption between the depression group and the control group (*p* < 0.05). Moreover, there were notable differences in diastolic blood pressure, BMI, waist circumference, total cholesterol, uric acid level, and sleep duration between the depression group and the control group (*p* < 0.05).

**Table 1:** Comparison of baseline data between the depression group and the control group

	Overall	Control group	Depression group	*p*
Age (years, mean ± SD)	66.57 ± 9.28	66.38 ± 9.36	66.89 ± 9.13	0.008
Gender (*n*, %)	Male: 4,562 (46.8)	Male: 3,185 (52.5)	Male: 1,377 (37.3)	0.000
	Female: 5,187 (53.2)	Female: 2,876 (47.5)	Female: 2,311 (62.7)	
Education (*n*, %)	No formal education (illiterate): 1,904 (19.5)	No formal education (illiterate): 991 (16.4)	No formal education (illiterate): 913 (24.8)	0.000
	Did not finish primary school: 2,101 (21.6)	Did not finish primary school: 1,139 (18.8)	Did not finish primary school: 962 (26.1)	
	Sishu/home school: 7 (0.1)	Sishu/home school: 5 (0.1)	Sishu/home school: 2 (0.1)	
	Elementary school: 2,360 (24.2)	Elementary school: 1,470 (24.3)	Elementary school: 890 (24.1)	
	Middle school: 2,280 (23.4)	Middle school: 1,622 (26.8)	Middle school: 658 (17.8)	
	High school: 754 (7.7)	High school: 554 (9.1)	High school: 200 (5.4)	
	Vocational school: 199 (2.0)	Vocational school: 156 (2.6)	Vocational school: 43 (1.2)	
	Two-/three-year college/associate degree: 86 (0.9)	Two-/three-year college/associate degree: 73 (1.2)	Two-/three-year college/associate degree: 13 (0.4)	
	Four-year college/bachelor’s degree: 54 (0.6)	Four-year college/bachelor’s degree: 49 (0.8)	Four-year college/bachelor’s degree: 5 (0.1)	
	Master’s degree: 3 (0.0)	Master’s degree: 1 (0.0)	Master’s degree: 2 (0.1)	
	Doctoral degree/Ph.D.: 1 (0.0)	Doctoral degree/Ph.D.: 1 (0.0)	Doctoral degree/Ph.D.: 0 (0.0)	
Marital status (*n*, %)	Married and live with spouse: 8,003 (82.1)	Married and live with spouse: 5,150 (85.0)	Married and live with spouse: 2,853 (77.4)	0.000
	Married but don’t living with spouse temporarily for reasons such as work: 529 (5.4)	Married but don’t living with spouse temporarily for reasons such as work: 287 (4.7)	Married but don’t living with spouse temporarily for reasons such as work: 242 (6.6)	
	Separated, don’t live together as a couple anymore: 19 (0.2)	Separated, don’t live together as a couple anymore: 10 (0.2)	Separated, don’t live together as a couple anymore: 9 (0.2)	
	Divorced: 101 (1.0)	Divorced: 59 (1.0)	Divorced: 42 (1.1)	
	Widowed: 1053 (10.8)	Widowed: 538 (8.9)	Widowed: 515 (14.0)	
	Never married: 44 (0.5)	Never married: 17 (0.3)	Never married: 27 (0.7)	
Blood pressure (systolic) (mmHg, mean ± SD)	129.63 ± 20.41	129.92 ± 20.38	129.14 ± 20.43	0.068
Blood pressure (diastolic) (mmHg, mean ± SD)	76.80 ± 12.21	77.10 ± 12.07	76.30 ± 12.42	0.002
BMI (kg/m^2^)	24.08 ± 3.62	24.16 ± 3058	23.95 ± 3.68	0.005
Waist circumference (cm, mean ± SD)	85.61 ± 12.94	86.15 ± 12.58	84.72 ± 1.46	0.000
Triglycerides (mg/dl, mean ± SD)	143.10 ± 91.32	143.97 ± 92.05	141.67 ± 90.11	0.227
Total cholesterol (mg/dl, mean ± SD)	183.45 ± 36.01	182.76 ± 35.37	184.59 ± 37.00	0.015
Glucose (mg/dl, mean ± SD)	102.66 ± 33.19	102.68 ± 32.16	102.62 ± 34.82	0.934
Uric acid (mg/dl, mean ± SD)	4.91 ± 1.37	5.00 ± 1.38	4.76 ± 1.36	0.000
Sleep (hours mean ± SD)	6.21 ± 1.92	6.54 ± 1.72	5.89 ± 2.12	0.000
Alcoholic beverage	More than once a month: 2586 (26.5)	More than once a month: 1834 (30.3)	More than once a month: 752 (20.4)	0.000
	Less than once a month: 739 (7.6)	Less than once a month: 474 (7.8)	Less than once a month: 265 (7.2)	
	None: 6424 (65.9)	None: 3753 (61.9)	None: 2671 (72.4)	

Note: BMI: body mass index. *p* < 0.05 was considered statistically significant.

### 3.3 Correlation analysis of depression and various indicators

We conducted correlation analyses (Table 2), which included variables such as age, BMI, blood pressure, pulse, waist circumference, sleep duration, and biochemical indicators. We found several variables to be associated with depression, including age, BMI, diastolic blood pressure, waist circumference, total cholesterol, uric acid, and sleep duration. There was a positive correlation between age and depression scores; depression is more likely to occur with age. Total cholesterol was also positively correlated with depression scores. BMI, blood pressure (diastolic), waist circumference, uric acid, and sleep duration were all indices that were negatively correlated with depression scores.

**Table 2:** Correlation analysis of depression and various indicators

Depression		
	*r*/*r*_s_	*p*
Age	0.039^**^	0.000
BMI	−0.038^**^	0.000
Blood pressure (systolic)	−0.018	0.068
Blood pressure (diastolic)	−0.036^**^	0.000
Pulse	0.015	0.130
Waist circumference	−0.062^**^	0.000
Triglycerides	−0.011	0.271
Total cholesterol	0.031^**^	0.002
Glucose	0.000	0.969
Uric acid	−0.098^**^	0.000
Sleep	−0.266^**^	0.000

Note: BMI: body mass index. *p* < 0.05 was considered statistically significant.

** represents *p* < 0.05.

### 3.4 Multivariate logistic regression analysis of depression and related variables

We incorporated age, gender, waist circumference, and other variables to construct multivariate logistic regression equations (Table 3). The results revealed that women had a higher risk of depression in middle and old age compared to men, and this difference was statistically significant (OR = 1.277, 95% CI 1.143–1.426, *p* < 0.05). Additionally, an increase in waist circumference was associated with a decreased risk of depression in middle-aged and elderly individuals, and this difference was statistically significant (OR = 0.995, 95% CI 0.990–0.999, *p* < 0.05). Moreover, an increase in sleep duration was found to be associated with a decreased risk of depression in middle-aged and elderly individuals, and this difference was statistically significant (OR = 0.802, 95% CI 0.783–0.821, *p* < 0.05). Furthermore, an improvement in educational level was associated with a decreased risk of depression in middle-aged and elderly individuals, and this difference was statistically significant (OR = 0.855, 95% CI 0.834–0.877, *p* < 0.05). Moreover, a worse marital status was found to increase the risk of depression in middle-aged and older adults, and this difference was statistically significant (OR = 1.104, 95% CI 1.068–1.142, *p* < 0.05). Lastly, a decreased frequency of alcohol consumption was associated with an increased risk of depression in middle-aged and older adults, and this difference was statistically significant (OR = 1.144, 95% CI 1.081–1.210, *p* < 0.05).

**Table 3:** Multivariate logistic regression analysis of depression and related variables

Variable	*b*	Standard error	Wald Chi-square value	*p*	OR	95% CI
Gender	0.244	0.057	18.607	0.000	1.277	1.143–1.426
Age	−0.004	0.003	1.804	0.179	0.996	0.991–1.002
BMI	−0.001	0.009	0.026	0.973	0.999	0.982–1.016
Blood pressure (systolic)	−0.002	0.001	2.017	0.156	0.998	0.995–1.001
Blood pressure (diastolic)	0.001	0.002	0.350	0.554	1.001	0.997–1.006
Pulse	0.000	0.002	0.011	0.917	1.000	0.996–1.004
Waist circumference	−0.005	0.002	4.922	0.027	0.995	0.990–0.999
Triglycerides	0.000	0.000	0.001	0.971	1.000	0.999–1.001
Total cholesterol	0.000	0.001	0.504	0.478	1.000	0.999–1.002
Glucose	0.001	0.001	0.608	0.436	1.001	0.999–1.002
Uric acid	−0.023	0.019	1.519	0.218	0.977	0.942–1.014
Sleep	−0.221	0.012	348.897	0.000	0.802	0.783–0.821
Education	−0.156	0.013	145.780	0.000	0.855	0.834–0.877
Marital status	0.099	0.017	33.712	0.000	1.104	1.068–1.142
Alcoholic beverage	0.134	0.029	21.896	0.000	1.144	1.081–1.210

Note: BMI: body mass index. *p* < 0.05 was considered statistically significant.

## 4 Discussion

Current theories of pathophysiology of depression include the monoamine theory and the hypothalamic–pituitary–adrenal (HPA) axis hyperactivity hypothesis, which states that monoamine neurotransmitter deficiencies and stressful events are responsible for the development of depressive episodes [15]. Studies have found that adults and adolescents with depression have different neurological changes; that is, depression has age-specific neurological changes [16]. To further understand the association between age, gender, and depression in older adults, a study was conducted, expanding the age range to include individuals aged 45 years and older for data analysis. Comparisons between the depression group and the control group revealed differences in age and sex. Correlation analysis further supported the significance of age in relation to depression. Multivariate logistic regression analysis indicated that gender is related to the high risk of depression in middle-aged and elderly individuals, while age itself does not appear to be a significant factor. Our study suggested that the occurrence of depression in middle-aged and elderly people in China is still related to gender, and it is more significant in women. Studies have found that women with depression are more likely to have symptoms than men, and at the same time, they are accompanied by an increase in inflammatory indicators, and some inflammatory indicators are not obvious in male patients with depression [17]. In further proteomic studies, it was found that the main biological processes in which female depression is affected by the relevant proteins are related to immune inflammation control [18]. The treatment of depression is still a difficult question to be studied, and recent studies have found that deep brain stimulation is more effective for women than for men [19]. Current research shows that women’s gender is associated with a risk of depression. The reason for this is that the immune system dysfunction associated with depression appears to be gender-specific, so many drug anti-inflammatory drugs exhibit antidepressant effects, and there are gender differences, but there are few studies on this gender difference, and sex hormones are an important factor in this gender difference [20].

Depression and obesity are two significant public health concerns that are currently receiving considerable attention. Some scholars have proposed that there are common biological pathways linking the two conditions, including genetic factors, metabolic factors, and emotional regulation [21]. Researchers believe that depression is related to obesity, and depressed people are more inclined to be obese, which leads to poor eating habits and lifestyles [22]. Several studies have found a correlation between depression and obesity or BMI. However, it has also been suggested that the reverse effect may be true, meaning that depression could lead to increased food intake and subsequent obesity [23]. In our study, we also examined the relationship between depression and obesity in middle-aged and elderly individuals over the age of 45 in China. We found that both BMI and waist circumference were associated with depression. However, BMI was not identified as an important correlated factor for depression after considering other relevant factors, and an increase in waist circumference was found to be associated with a reduced risk of developing depression. Obesity and depression go both ways, with altered gut microbiomes in obese individuals leading to systemic inflammation and mood disorders [24]. Further studies found that the recurrence of depression received gender modification, with recurrence of depression in women positively correlated with obesity-related traits, and in men, both recurrence of depression and subcutaneous fat in men [25]. This finding helps to explain why BMI was associated with depression in our study, while not being identified as a risk factor for depression. Overall, the relationship between depression and obesity is complex and multifaceted. Further research is needed to better understand the underlying mechanisms and to develop effective interventions for individuals affected by both conditions. Obesity and depression have been found to be closely related, with each potentially exacerbating the other. However, it is important to note that this does not necessarily imply a causal relationship between weight and depression. Instead, obesity and depression are often considered comorbidities, meaning that they frequently occur together but are not directly linked. These comorbidities can be attributed to bidirectional relationships and common genetic factors [26]. Interestingly, our study discovered that an increased waist circumference actually reduces the risk of depression, which brings to mind the “Jolly Fat hypothesis” which is that higher BMI is significantly associated with better mental health conditions and events, and some studies have suggested that depression is inversely correlated with BMI in older women [27]. This finding contradicts some previous studies that have suggested a positive correlation between depression and central obesity. The discrepancy in results may be due to differences in the racial and genetic makeup of the populations included in the studies. It is also worth noting that our study only assessed depression through scoring, while other studies may have included participants who were receiving medication for depression. These factors could contribute to the conflicting results. Given the current state of research, it is more appropriate to study obesity and depression as comorbidities rather than as directly interrelated conditions.

Through questionnaires, we have discovered that participants’ level of education, marital status, alcohol consumption, and nighttime sleep duration are all factors associated with depression. Specifically, we found that individuals with lower education levels, worse marital statuses, and shorter sleep durations are at a higher risk of experiencing depression. The study found that both culture and income were associated with the aggravation of depression, and the higher the level of education and the lower the income, the more obvious the aggravation of depression [28]. For older adults, frailty and depression were positively correlated, with variables such as education level, income status, people living with them, regular medications, forgetting to take medications, urinary incontinence, hospitalization, and accidents found to influence frailty and depression [29]. Another study is consistent with our findings, suggesting that low educational attainment is associated with a risk of mental disorders, substance use disorders, and self-harm at all ages [30]. Marital status is a well-established potential risk factor for depression, but analysis across multiple countries suggests that unmarried people may be at greater risk of depression, and it is also related to gender and educational attainment [31]. Studies in older adults have found a correlation between reduced sleep and depression, and early detection of depressive states can predict future sleep disorders [32]. To further explore the mechanism, Mendelian randomization provided genetic evidence for an individual’s sleep characteristics and increased risk of depression and found a causal relationship between insomnia and daytime naps and an increased risk of depression [33]. Our study has found that longer sleep duration is associated with a reduced risk of depression. However, it is important to note that excessive sleep, or even excessive drowsiness, can also serve as an early warning sign of depression [34]. The mechanism of the correlation between sleep disorders and depression includes several aspects: (1) it is related to the activation and inflammation of the HPA axis, which leads to a decrease in serotonin synthesis, which affects other factors involved in the pathophysiology of neuropsychiatric disorders; and (2) changes in the gut microbiota affect the microbiota–gut–brain axis [35]. Therefore, we can conclude that a longer sleep duration within an appropriate time frame is more beneficial in reducing the risk of depression. Interestingly, our study also found that drinking less alcohol increases the risk of depression. A longitudinal population study conducted in Sweden revealed that individuals who never drink alcohol have a higher risk of depression compared to light drinkers, while those who engage in risky drinking have a higher risk of depression compared to non-risk drinkers [36]. In our study, the highest level of drinking was considered light drinking, as it occurred more than once a month, and the results were consistent with this finding. However, some researchers have suggested that moderate drinking does not have a clear protective association with depression in old age. It is worth noting that inaccurate reporting of the specific amount of alcohol consumed may contribute to this discrepancy [37]. Furthermore, there appears to be a bidirectional relationship between alcohol consumption and depression, as comorbidities of depression and alcohol addiction are frequently observed clinically. Depression can lead to increased alcohol consumption, and increased alcohol consumption may induce depression. The mechanism behind this relationship may be related to the activation and decline of hippocampal microglia [38]. A study in the United States found that different drinking frequencies had different effects on depression, and smoothed curve fitting revealed for the first time an “M-shaped” relationship between drinking frequency and depression [39]. In summary, adopting healthy lifestyle habits is crucial in reducing the risk of depression. Our research did not find a significant correlation between blood pressure, blood lipids, blood sugar, and the risk of depression in middle-aged and elderly individuals. However, there are many factors that affect depression. Studies have found that the residential environment can affect depression, and residential greening can effectively reduce the impact of other factors on depression and have a role in supporting mental health [40]. Socially isolated environmental conditions can also exacerbate depression, leading to a high incidence of depressive events [41]. Studies have found that the environment in which you live at a given age explains the psychological effects, so both the living environment and the living experience have an impact on depression [42].

Our study had several limitations that should be acknowledged. First, the grouping of depression was based on the current epidemiological general scale, which relies on subjective responses from the subjects. As a result, the accuracy of the depression diagnosis may not be sufficient. Second, there was a certain level of subjectivity in the questionnaire responses, leading to potential arbitrariness and a significant amount of data loss. This could have introduced certain inaccuracies in our findings. Third, our study did not include data on smoking, exercise, environment and chronic diseases, which could have influenced the results. The absence of these variables might have contributed to differences in the outcomes observed. It is important to note that our study utilized data from public databases. Fourth, this is a cross-sectional study, so causality cannot be accounted for. In future research, we plan to establish multicenter cohorts to gather more comprehensive data and conduct targeted and in-depth studies.

## 5 Conclusion

Depression is now widely acknowledged as a significant public health issue, and its prevalence among middle-aged and elderly individuals in China is on the rise. Various factors such as gender, waist circumference, sleep duration, education, marital status, and drinking habits have been found to be associated with depression in this population. Specifically, women, low education level, and experiencing dissatisfaction in marriage are connected with the high risk of depression among middle-aged and elderly individuals. On the other hand, increased waist circumference, moderate alcohol consumption, and longer sleep duration are connected with the low risk of depression.
